# A comparative study of *Mycobacterium avium *subsp. *avium *and *Mycobacterium avium *subsp. *hominissuis *in experimentally infected pigs

**DOI:** 10.1186/1746-6148-8-11

**Published:** 2012-01-27

**Authors:** Angelika Agdestein, Tone B Johansen, Øyvor Kolbjørnsen, Anne Jørgensen, Berit Djønne, Ingrid Olsen

**Affiliations:** 1 Norwegian Veterinary Institute, PO. Box 750 Sentrum, N-0106 Oslo, Norway; 2 Norwegian Pig Health Service, Animalia, PO. Box 396 Økern, N-0513 Oslo, Norway

**Keywords:** *Mycobacterium avium*, Experimentally infected pigs, Transmission, Source of infection

## Abstract

**Background:**

*Mycobacterium avium *subsp. *avium *(*Maa*) and *Mycobacterium avium *subsp. *hominissuis *(*Mah*) are opportunistic pathogens that may infect several species, including humans and pigs. *Mah *is however more frequently isolated from pigs than *Maa*, and it is unclear if this is due to difference in virulence or in exposure to the two organisms. Clinical isolates of each subspecies were administered perorally to ten domestic pigs, respectively. The animals were sacrificed at six and 12 weeks after inoculation. At necropsy, macroscopic lesions were recorded, and tissue samples were collected for mycobacterial culture, IS*1245 *real time PCR and histopathological examination. Culturing was also performed on faecal samples collected at necropsy.

**Results:**

Macroscopic and histopathological lesions were detected in pigs infected with each subspecies, and bacterial growth and histopathological changes were demonstrated, also in samples from organs without gross pathological lesions. Six weeks after inoculation, live *Mah *was detected in faeces, as opposed to *Maa*. The presence of live mycobacteria was also more pronounced in *Mah *infected tonsils. In comparison, the *Maa *isolate appeared to have a higher ability of intracellular replication in porcine macrophages compared to the *Mah *isolate.

**Conclusions:**

The study shows that both subspecies were able to infect pigs. Additionally, the more extensive shedding of *Mah *might cause pig-to-pig transmission and contribute to the higher incidence of infection caused by this subspecies.

## Background

The genus *Mycobacterium *covers a broad spectrum of acid-fast staining species, ranging from harmless saprophytes to significant pathogens [1]. *M. avium *subsp. *avium *(*Maa*) and *M. avium *subsp. *hominissuis *(*Mah*) are two subspecies of *M. avium*, subordinated the *Mycobacterium avium *complex (MAC) [2]. *Mah *can cause serious systemic infection in immunocompromised patients, such as humans infected with HIV [3]. Additionally, this opportunistic pathogen can cause cervical lymphadenitis in children and lung infections in patients with underlying lung disease [1]. In pigs, *Mah *is frequently responsible for lesions in lymph nodes of the digestive tract, and can also lead to systemic infection with affection of parenchymatous organs. Since *Mah *usually does not cause clinical signs in pigs, the lesions are mainly detected at slaughter [4]. *Maa *is the causative agent of tuberculosis in birds, in which it acts as an obligate pathogen, causing contagious, chronic disease [5,6]. *Maa *has also been isolated from humans and pigs with mycobacteriosis [7,8].

Before molecular typing enabled differentiation of *Maa *and *Mah *isolates, birds were believed to be an important source of mycobacteriosis in pigs [9]. However, various molecular investigations on clinical isolates have shown this is rather the exception than the rule [7,10,11]. Although *Maa *has been reported in pigs and in humans [7,8], *Mah *plays the key role in MAC infections in these mammalian species [12-16]. In Norway only *Mah *has been detected in pigs [17]. Peat and sawdust have proved to be important sources of porcine *Mah *infection by various authors [11,18-20], whereas human infection often is contracted from water [6,21]. The cause of the discrepancies of the prevalence of each subspecies with regards to hosts remains unclear. Suggested factors are sampling procedures, differences in farming conditions and environmental exposure [8]. Differences between *Maa *and *Mah *with regards to virulence and susceptibility in pigs might also be important factors contributing to the dominant presence of *Mah*. Infections with *M. avium *might be of zoonotic importance [4,22], and the possibility of pigs being a source for human infection cannot be ruled out [10,16]. More knowledge about routes of transmission in animals and humans is required for control of the disease [3,13].

Experimental infection of pigs with *M. avium *prior to the division of subspecies *avium *into *Maa *and *Mah *has been described in the literature [9,23-27]. After the application of the new systemic nomenclature, *Maa *infection has been characterized in wild boars by experimental infection [28]. Additionally, collection strains of *Maa *and *Mah *were compared in a study aiming to optimise diagnostic methods [29].

The aim of the present study was to elucidate whether *Maa *and *Mah *have a different potential to infect pigs that may explain the differences in prevalence between the two subspecies. The results showed a slightly, but not significantly, elevated ability of *Mah *to infect pigs, compared to *Maa*. The main finding, however, was the event of faecal shedding of *Mah *at 6 weeks after infection, which was not observed in pigs infected with *Maa*.

## Methods

### Animals

The use of experimental animals was approved by the Norwegian Animal Research Authority. Twenty Duroc/Norwegian Landbreed crossbred pigs (*Sus scrofa*) were bought from a commercial pig farm, from which mycobacteriosis had not been reported. Random sampling for bacteriological analysis of peat and sawdust used at the farm was completed, and mycobacteria were not detected. To further exclude the presence of *M. avium *in the herd, bacteriological examination of *Lymphonodii *(*Lnn*). *mandibulares, Lnn. jejunales, Lnn. ileocolici, Lnn. colici*, tonsils, spleens and Peyer's patches of jejunum and ileum collected from animals from the same farm at conventional slaughter was performed. No mycobacteria were detected. Organs from these un-inoculated animals were also used for macroscopic and microscopic comparison in the pathological examination of organs from the experimentally infected animals. Due to the limited housing facilities a formal negative control group held under identical conditions as the inoculated animals was not possible to include in the study. As the study was aiming at comparing two subspecies, such a control group was not considered necessary as long as the original herd was to the authors' best knowledge uninfected.

### Experimental design

At the age of 6 weeks, the pigs to be inoculated were housed in two groups of ten at a research facility at the Norwegian Veterinary Institute. Between arrival at the research facility and inoculation, the animals were allowed one week of acclimatization. The two groups were evenly distributed with regards to weight, sex and litter origin, and kept in separate rooms, each accessible only through sluice chambers. Water was provided from a common drinking water system, from which no mycobacteria were detected by culture. The animals were fed commercially available pig feed and kept on a bedding of wood shavings, which is considered less likely to harbour mycobacteria, as opposed to sawdust [11].

The pigs were inoculated per os with 5 × 10^9 ^viable bacteria at the age of 7 weeks. Animals #1 - #10 received an isolate of *Mah*, and animals #11 - #20 an isolate of *Maa*. The inocula were mixed with raspberry jam intended for human consumption and applied at the back of the tongue. The animals had been trained for a week in advance to efficiently swallow treats applied in this manner.

Five pigs from each group of ten were euthanized under general anaesthesia for pathological and bacteriological examination at 6 weeks after inoculation, the remaining pigs at 12 weeks. Prior to euthanasia, infection was confirmed by IFN-γ assays and tuberculin testing. One animal in the last group to be sacrificed was euthanized after 8 weeks, as its weight gain was unsatisfactory. General anaesthesia of the pigs was achieved by deep i.m. injection of xylazine 2 mg/kg (Rompun^® ^vet. 20 mg/ml, Bayer, Oslo, Norway), butorphanol 0.2 mg/kg (Dolorex^® ^vet. 10 mg/ml, Intervet/Shering Plough Animal Health, Bergen, Norway) and ketamine 15 mg/kg (Ketalar^® ^50 mg/ml, Pfizer, Oslo, Norway) prior to euthanization by the i.v. administration of 200 mg/kg pentobarbital (Pentobarbital 10%, NAF Apotek, Oslo). Gross pathological examination and sampling was performed immediately post mortem.

### Preparation of bacterial inoculum

Two Norwegian clinical isolates of *M. avium*, VI101 and 1794, were used in the study. VI101, a smooth and opaque *Mah *isolate, originates from a porcine cervical lymph node with pathological lesions detected at slaughter. 1794 is a rough *Maa *isolate from a Norwegian Rough-legged Buzzard (*Buteo lagopus*) diagnosed with avian tuberculosis. Data on isolation, restriction fragment length polymorphism (RFLP) type and biofilm producing abilities for both isolates were presented by Johansen et al. 2007 and 2009 [17,30].

Bacterial inocula were prepared from seven days subcultures grown on plates of Middlebrook 7H10 (BD Diagnostics, Sparks MD) with 10% OADC (BD Diagnostics) at 37°C. For adjusting the number of bacteria in the inoculum, real time PCR on the single copy housekeeping gene *hsp*65 [31] was performed on serial dilutions of a suspension adjusted to McFarland standard 2.0, homogenised through a 23G needle. Diluents were HBSS (Invitrogen, Oslo, Norway) in cellular assays and MQ water in animal assays. To confirm the real time PCR results, the dilutions were additionally seeded onto Middlebrook 7H10 (BD Diagnostics) and incubated at 37°C for 2 weeks for CFU count.

### Macrophage infection assay

Peripheral blood mononuclear cells (PBMCs) were separated by density gradient centrifugation from 50 ml of heparinised blood from four non-inoculated pigs at the original farm. Summarized, 2 × 25 ml blood was layered on top of 2 × 10 ml Lymfoprep (Axis-Shield, Oslo, Norway) and centrifuged at 400 g for 30 min, followed by washing three times. The cells were resuspended in 50 ml RPMI-1640 with sodium bicarbonate (Sigma-Aldrich, Oslo, Norway) and 1% L-glutamine (Sigma-Aldrich) at a concentration of 3.5 × 10^6 ^PBMCs per ml. One ml of cell suspension was added to each well of 12 well cell culture plates (Sigma-Aldrich), giving 3.5 × 10^5 ^monocytes per well. To isolate adherent cells, the plates were incubated at 37°C in 0.5% CO_2 _for one hour, followed by two steps of washing with pre-warmed medium to remove lymphocytes. The cells were incubated further for one week in medium with 10% swine serum (SS) (Rockland Immunochemicals Inc., Gilbertsville, PA) to mature the monocytes into macrophages. Every second day 0.5 ml of the supernatant was carefully replaced with fresh medium. The seven days old macrophage cultures were infected with a multiplicity of infection (MOI) of 10:1 bacteria per macrophage, as determined by *hsp*65 real time PCR. Macrophages from all four pigs were infected in duplicate wells with *Mah *and *Maa*, respectively. After six hours of incubation, the wells were washed vigorously three times with medium to remove any extracellular bacteria, and incubated further. Cell lysis was performed in 1.5 ml MQ water. Lysates were harvested from both infected and uninfected control wells after six hours, and after one and seven days of infection and stored at -20C in 2 ml O-ring vials (Biospec Products Inc., Techtum Lab. Umeå, Sweden) containing 200 μl 0.1 mm silica beads (Biospec Products Inc.). After completion of sampling, lysates were thawed, inactivated at 100°C for 20 min, bead beaten and analysed by *hsp*65 real time PCR for determination of the extent of intracellular replication [31,32].

### Immunological testing

All animals were tested for IFN-γ response to purified protein derivate from *M. avium *(PPDa) at 5 weeks, and eight of the remaining pigs at 11 weeks. Briefly, one ml of heparinised blood was stimulated overnight in 5% CO_2 _at 37°C with PPDa or PBS as negative control. Plasma was harvested and stored at -70C until analysed, using a commercial IFN-γ swine ELISA kit (Invitrogen) according to the manufacturer's recommendations.

Tuberculin skin test was performed before euthanasia, after blood sampling for IFN-γ testing was completed. In-house produced PPDa from *Mah *D4 and PPDt from pooled *M. tuberculosis *strains E9655 and E5 were injected intradermally behind respectively left and right ear of the animals at a dose of 0.1 ml. The thickness of the skin was measured before and 72 h after injection. Increased measures and occurrence of visible changes in the skin were recorded. Any visible oedema or redness or a > 2 mm increase of the skin thickness at the injection site was regarded as a positive reaction of delayed hypersensitivity to PPD.

### Gross pathology and histopathology

The animals were successively euthanized and placed on a table covered in plastic. A thorough post mortem examination was performed immediately after euthanasia. The table was disinfected and the cover changed between the animals. Incisions were made on a selection of lymph nodes and organs, in order to reveal potential macroscopic lesions compatible with mycobacterial infection, which were defined as distinct foci of white-yellow tissue, further addressed as tuberculous lesions. A standardized sampling procedure was performed on tonsils, the medial lung lobe, the dorsal end of the spleen, the right medial liver lobe, jejunal and ileal Peyer's patches, *Lnn. mandibulares, Lnn. bifurcationis sinn*., and the gastrointestinal lymph nodes *Lnn. jejunales, Lnn. ileocolici *and *Lnn. colici *in each animal. Each tissue sample was subjected to histopathological and bacteriological examination. Five grams of faeces were collected at necropsy from each inoculated pig. Gloves, scalpels and cutting boards were changed between each sampling, to avoid cross contamination between organs and animals.

Tissues for light microscopy were fixed in phosphate-buffered 4% formaldehyde, prior to routine processing and paraffin embedding, followed by sectioning at 3-4 μm and staining with hematoxylin-eosin (HE) and Ziehl-Neelsen (ZN) to facilitate the detection of acid-fast bacteria. An additional section of those samples indicative of fibrous tissue were stained with van Gieson (vG) and samples indicative of mineralisation were stained with von Kossa. On microscopy, granulomatous lesions were classified into three categories according to the system described by Hibiya et al. [33]. However, the classification system was slightly simplified, due to the wide range of variations within the histopathological appearance. In the present study, the term exudative was applied on unencapsulated lesions ranging from the mere presence of single multinucleated giant cells and/or a few macrophages to the larger, well-circumscribed granulomas consisting of epitheloid cells and various numbers of lymphoid cells, multinucleated giant cells, neutrophils and eosinophils. Reactions were classified as proliferative when the lesions were partially or fully surrounded by fibroblasts and showing various degrees of encapsulation, from loosely woven connective tissue to thick collagen capsules. Sections with the simultaneous occurrence of exudative and proliferative reactions were classified as mixed granulomas. Necrosis and calcification could occur within all the categories.

All sections presenting multinucleated giant cells and granulomatous lesions were examined for porcine circovirus 2 (PCV2) by immunohistochemical staining, since PCV2 is a common differential diagnose to mycobacteriosis when granulomatous reactions are observed in pigs [34]. Briefly, after clearing with xylene, sections were rehydrated and stained with a monoclonal mouse anti-PCV2 antibody (mAb F1217-2C6 H9 A2) at dilution 1:20 000. The antibody was kindly provided by Dr. Gordon Allan, Department of Agriculture for Northern Ireland, Veterinary Science Division, Belfast, UK. PCV2 was not detected in any samples.

### Bacteriological examination

Defined amounts of tissue and faecal material were decontaminated as previously described [35]. Briefly, 1-3 g of the sample were homogenised in saline, decontaminated with 5% oxalic acid with 0.1% malachite green and centrifuged, followed by resuspension of the pellet in saline and inoculation on slants of Middlebrook 7H10 medium (BD Diagnostics) with 10% OADC (BD Diagnostics) with and without antibiotics (final concentrations of 100 μg/ml carbenicillin, 200 U/ml polymyxin B sulphate, 19.5 μg/ml trimethoprim lactate and 10 μg/ml amphotericin B), Petragnani's medium (BD Diagnostics), Stonebrink's medium (BD Diagnostics) and Loewenstein-Jensen's egg-based medium (BD Diagnostics). An additional step of decontamination with NaOH prior to the step of treatment with oxalic acid with malachite green was performed on faecal samples. The media were incubated for 8 weeks at 37°C and observed for growth on a weekly basis. Cultures of acid-fast bacteria were further identified by IS*1245 *RFLP [17], IS*901 *PCR (primer 901a and 901c) [36] and IS*1245 *PCR (primer p40 and p41) [37] to confirm the expected subspecies and RFLP profile and thereby rule out cross contamination.

For real time PCR, template DNA was extracted from tissue samples by using NucliSENS^® ^easyMag^® ^(Biomérieux, Marcy l'Etoile, France). Briefly, 0.1 ml of the saline used for overnight soaking of the sample homogenate was transferred to 1 ml NucliSENS^® ^easyMag^® ^lysis buffer (Biomérieux) and inactivated at 80°C for 20 min prior to 2 min bead beating with 0.2 ml of 0.1 mm silica beads (Biospec Products Inc.), followed by DNA extraction by the generic protocol. Real time PCR was performed on the template DNA by amplification of a 82 bp target sequence of the insertion element IS*1245*, as described by Agdestein et al. [20]. Samples were regarded as positive when Ct ≤ 38.

### Statistical analysis

Differences between the groups were assessed by using a Wilcoxon-Mann Whitney non-parametric test. *P *≤ 0.05 was considered statistically significant.

## Results

### Clinical signs

Animal #15 and #18, both inoculated with *Maa*, had diarrhea for five and two days, respectively, starting three days after infection. Animal #18 developed a cough, which was later explained by eosinophilic interstitial pneumonia detected at pathological examination, considered unrelated to mycobacterial infection. Animal #6, inoculated with *Mah*, showed a decrease in growth rate and was therefore euthanized and subjected to necropsy at week eight, upon which no macroscopic lesions compatible with mycobacteriosis were detected. The remaining animals did not show any clinical signs of infection.

### Immunological testing

To confirm the infection, the animals were tested in an IFN-γ assay and the tuberculin test. All animals were negative in the IFN-γ assay before inoculation, while after 5 weeks, samples from eight (40%) out of the infected animals produced IFN-γ in response to PPDa. Of these, five were infected with *Mah*, and three with *Maa*. All inoculated animals tested at 11 weeks after infection, showed a strong IFN-γ response to PPDa, and there was no significant difference between animals in the *Mah *and *Maa *groups. Positive reactions on tuberculin skin testing were seen in all infected groups, but not in every individual. The strongest reactions were seen at 11 weeks, in both groups. Ulcerations or necrosis of the skin were observed in six animals. Three of the animals tested at 5 weeks showed no reaction to either types of PPD used. One of these pigs was infected with *Mah*, the other two with *Maa*.

### Macrophage infection assay

**Figure 1:**
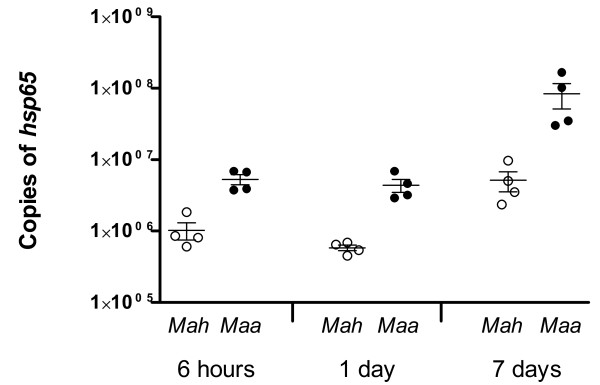
**Replication of *Mycobacterium avium *subsp**. *hominissuis *(*Mah*) or *Mycobacterium avium *subsp. *avium *(*Maa*) in porcine macrophages. Intracellular replication in macrophages from four un-inoculated pigs was measured by the number of the single copy housekeeping gene *hsp*65 copies, as determined by real time PCR analysis of lysates harvested at six hours, one day and seven days after infection. Numbers are presented as the mean of duplicate wells. Control lysates were negative on real time PCR and are not shown.

Both *Mah *and *Maa *were able to infect and replicate within macrophages, however some differences between the subspecies were observed. *Maa *showed a greater ability of invasion and replication within porcine macrophages at all sampled points in time (Figure 1). The most prominent difference was observed at day seven.

### Gross pathology and histopathology

Macroscopic lesions compatible with mycobacterial infection were found in six animals (Table 1), ranging from one to four mm in size. The majority of macroscopic lesions were seen in mesenteric lymph nodes. In the animals sampled at week six, visible tuberculous lesions were detected only in one animal, which was inoculated with *Maa*. At 12 weeks, 10 samples from three animals in the *Mah *group and three samples from two animals in the *Maa *group, showed tuberculous lesions on macroscopic examination. Only two *Lnn. mandibulares*, both from pigs inoculated with *Mah*, had visible tuberculous lesions. There were no visible tuberculous lesions in tonsils, spleens, *Lnn. bifurcationis sinn*., nor in the lungs in any of the animals. Crater-like changes of four mm were seen in the jejunal Peyer's patches of animals #18 and #19, inoculated with *Maa*.

**Table 1:** Pathological and bacteriological findings in pigs inoculated with *Mycobacterium avium *subsp.

	*Mycobacterium avium *subsp. *hominissuis*																																							
**Time after inoculation**	**6 weeks**																				**8 weeks**				**12 weeks**															

**Animal no**	**#1**				**#2**				**#3**				**#4**				**#5**				**#6**				**#7**				**#8**				**#9**				**#10**			

	**m**	**h**	**c**	**p**	**m**	**h**	**c**	**p**	**m**	**h**	**c**	**p**	**m**	**h**	**c**	**p**	**m**	**h**	**c**	**p**	**m**	**h**	**c**	**p**	**m**	**h**	**c**	**p**	**m**	**h**	**c**	**p**	**m**	**h**	**c**	**p**	**m**	**h**	**c**	**p**

*Lnn. mandibulares*	-	-	+	+	-	-	+	-	-	-	+	-	-	-	+	+	-	-	+	-	-	-	+	+	+	+*	+	-	-	+	+	+	+	+*	+	+	-	+*	+	+

Tonsils	-	+*	+	+	-	-	+	+	-	-	+	+	-	-	+	+	-	-	+	+	-	-	+	-	-	-	+	-	-	-	C	+	-	+*	+	+	-	+*	+	-

*Lnn. bifurcationis sinn*.	-	-	+	+	-	-	-	-	-	-	-	-	-	-	-	-	-	-	-	-	-	-	-	-	-	-	-	-	-	-	-	-	-	-	+	+	-	-	-	+

Lung	-	-	-	-	-	-	-	-	-	-	-	-	-	-	-	-	-	-	-	-	-	-	-	-	-	-	-	-	-	-	-	-	-	+	-	nd	-	-	-	-

Spleen	-	-	-	-	-	-	-	-	-	-	-	-	-	-	-	-	-	-	-	-	-	-	-	-	-	-	-	-	-	-	-	+	-	-	-	nd	-	-	-	+

Liver	-	-	-	-	-	+	-	-	-	-	-	-	-	-	-	-	-	-	-	-	-	-	-	-	-	+	-	-	-	+	-	+	+	+*	-	nd	-	-	-	+

*Lnn. jejunales*	-	-	+	+	-	-	+	+	-	+*	+	+	-	-	+	+	-	+*	-	-	-	+*	+	+	+	+*	+	+	+	+*	+	+	+	+*	+	nd	-	+*	+	-

*Lnn. ileocolici*	-	-	+	-	-	-	+	+	-	-	-	-	-	-	+	-	-	-	-	-	-	-	+	-	+	+*	+	+	-	+*	+	+	+	+*	+	nd	-	+	-	-

*Lnn. colici*	-	-	+	-	-	-	+	+	-	-	-	-	-	-	-	-	-	-	-	-	-	-	+	-	-	-	-	-	+	+*	+	+	+	+*	+	nd	-	-	-	-

Jejunal Peyer's patches	-	-	+	-	-	-	-	-	-	-	-	-	-	-	+	+	-	-	+	-	-	-	-	nd	-	+	-	-	-	+	-	-	-	+	-	nd	-	-	-	-

Ileal Peyer's patches	-	-	+	+	-	-	+	-	-	-	+	-	-	+	+	+	-	-	-	-	-	-	+	nd	-	+	-	-	-	-	-	-	-	+*	-	nd	-	+	-	-

Faeces	nd	nd	+	nd	nd	nd	+	nd	nd	nd	+	nd	nd	nd	+	nd	nd	nd	-	nd	nd	nd	+	nd	nd	nd	-	nd	nd	nd	-	nd	nd	nd	-	nd	nd	nd	-	nd

	***Mycobacterium avium *subsp. *avium***																																							

**Time after inoculation**	**6 weeks**																			**12 weeks**																				

**Animal no**	**#11**			**#12**				**#13**				**#14**				**#15**				**#16**				**#17**				**#18**				**#19**				**#20**				

	**m**	**h**	**c**	**p**	**m**	**h**	**c**	**p**	**m**	**h**	**c**	**p**	**m**	**h**	**c**	**p**	**m**	**h**	**c**	**p**	**m**	**h**	**c**	**p**	**m**	**h**	**c**	**p**	**m**	**h**	**c**	**p**	**m**	**h**	**c**	**p**	**m**	**h**	**c**	**P**

*Lnn. mandibulares*	-	+*	+	+	-	+	+	-	-	-	+	-	-	-	-	-	-	-	+	-	-	-	+	-	-	-	+	+	-	+	+	-	-	+*	+	-	-	+*	+	+

Tonsils	-	+	-	+	-	-	-	-	-	-	-	-	-	-	-	+	-	+	-	-	-	-	-	-	-	-	-	-	-	-	-	-	-	+*	+	+	-	+*	+	+

*Lnn. bifurcationis sinn*.	-	-	-	-	-	-	-	+	-	-	-	-	-	-	-	-	-	-	-	-	-	-	-	-	-	-	-	-	-	+	-	-	-	+	-	-	-	-	-	+

Lung	-	-	-	-	-	-	-	-	-	-	-	+	-	+	-	-	-	-	-	-	-	-	-	-	-	-	-	+	-	-	-	+	-	-	-	+	-	+	-	+

Spleen	-	+	-	-	-	-	-	-	-	-	-	-	-	-	-	-	-	-	-	+	-	-	-	-	-	-	-	+	-	-	-	-	-	-	-	+	-	-	-	+

Liver	-	-	-	-	-	-	-	-	-	-	-	-	-	-	-	-	-	-	-	-	-	-	-	+	-	-	-	+	-	-	-	-	-	+	-	-	-	-	-	+

*Lnn. jejunales*	-	-	+	+	-	-	+	-	-	-	+	-	-	-	+	-	-	-	+	+	-	-	-	-	-	-	+	-	+	+*	+	-	+	+*	+	+	-	+	+	+

*Lnn. ileocolici*	+	+	+	+	-	-	+	+	-	-	-	-	-	-	+	-	-	-	+	-	-	-	-	-	-	-	-	-	-	+	+	-	+	+	+	+	-	-	+	-

*Lnn. colici*	-	-	-	+	-	-	+	-	-	+	-	-	-	-	-	-	-	-	+	+	-	-	+	-	-	-	+	-	-	-	-	+	-	-	-	-	-	-	+	-

Jejunal Peyer's patches	-	-	+	+	-	-	+	-	-	+	+	-	-	-	-	-	-	-	+	-	-	-	-	-	-	-	-	-	-	+	-	+	*	+	-	+	-	-	-	+

Ileal Peyer's patches	-	-	+	+	-	+*	+	-	-	-	+	+	-	-	-	-	-	-	+	-	-	+	+	+	-	-	-	-	-	+	+	-	-	+	-	+	-	+*	+	-

Faeces	nd	nd	-	nd	nd	nd	-	nd	nd	nd	-	nd	nd	nd	-	nd	nd	nd	-	nd	nd	nd	-	nd	nd	nd	-	nd	nd	nd	-	nd	nd	nd	-	nd	nd	nd	-	nd

*hominissuis *or *Mycobacterium avium *subsp. *avium*

m = macroscopic examination. Positive when visible tuberculous lesions are present

h = histopathological examination. Positive when granulomatous lesions present. * indicates the presence of acid-fast bacilli

c = cultural examination: Positive when acid-fast staining, characteristic colonies are present

p = real time PCR: Positive at Ct values ≤ 38

nd = not done or not applicable

C = contaminated

Additionally, on gross examination cystic changes of two to 15 mm were detected in 10 lymph node samples originating from six different animals in the *Maa *group. Only two of these samples had additional macroscopic lesions in the surrounding tissue. However, all samples with cystic changes, except one, were accompanied by either the presence of acid-fast rods on histopathological examination or positive results from culture or real time PCR The fluid content within the cystic compartments ranged from transparent to opaque and proved sterile on general bacteriologic examination. To the authors best knowledge the number of cystic lesions of lymph nodes reported previously in pigs is negligible, and cysts have not been described in association with mycobacterial disease in swine. To determine whether or not mycobacteria were directly responsible for the cystic changes, further investigations are required.

On histopathological examination granulomatous lesions were seen in 32 out of 110 (29%) standardized tissue samples from pigs inoculated with *Mah*, and acid-fast bacilli were detected in 20 (60%) of these. In pigs inoculated with *Maa*, granulomatous lesions were detected in 30 out of 110 (27%) samples, nine containing acid-fast bacilli (Tables 1 and 2). The majority of the lesions were classified as exudative, while encapsulated granulomas were always seen together with exudative lesions, and the samples therefore classified as mixed. Six samples from the *Mah *group and two samples from the *Maa *group were of the mixed type, and they were all found in gastrointestinal lymph nodes. One animal infected with *Maa *had ulceration of the mucosa in addition to exudative reaction in both samples from the Peyer's patches.

**Table 2:** Histopathological findings in pigs inoculated with *Mycobacterium avium *subsp.

	*Mycobacterium avium *subsp. *hominissuis*																			
**Time after inoculation**	**6 weeks**										**8 weeks**		**12 weeks**							

**Animal no**	**#1**		**#2**		**#3**		**#4**		**#5**		**#6**		**#7**		**#8**		**#9**		**#10**	

	**gl**	**n/c**	**gl**	**n/c**	**gl**	**n/c**	**gl**	**n/c**	**gl**	**n/c**	**gl**	**n/c**	**gl**	**n/c**	**gl**	**n/c**	**gl**	**n/c**	**gl**	**n/c**

*Lnn. mandibulares*	-	-/-	-	-/-	-	-/-	-	-/-	-	-/-	-	-/-	m*	+/+	e	-	e*	+/+	e*	-/-

Tonsils	e*	-/-	-	-/-	-	-/-	-	-/-	-	-/-	-	-/-	-	-	-	-	e*	+/-	e*	-/-

*Lnn. bifurcationis sinn*.	-	-/-	-	-/-	-	-/-	-	-/-	-	-/-	-	-/-	-	-	-	-	-	-	-	-/-

Lung	-	-/-	-	-/-	-	-/-	-	-/-	-	-/-	-	-/-	-	-	-	-	e	-	-	-/-

Spleen	-	-/-	-	-/-	-	-/-	-	-/-	-	-/-	-	-/-	-	-	-	-	-	-	-	-/-

Liver	-	-/-	e	-/-	-	-/-	-	-/-	-	-/-	-	-/-	e	-	e	-	e*	-	-	-/-

*Lnn. jejunales*	-	-/-	-	-/-	e*	-/-	-	-/-	e*	-/-	e*	-/-	m*	+/+	m*	+/+	m*	+/+	e*	-/-

*Lnn. ileocolici*	-	-/-	-	-/-	-	-/-	-	-/-	-	-/-	-	-/-	m*	+/+	m*	+/-	e*	+/-	e	-/-

*Lnn. colici*	-	-/-	-	-/-	-	-/-	-	-/-	-	-/-	-	-/-	-	-	m*	-	e*	+/+	-	-/-

Jejunal Peyer's patches	-	-/-	-	-/-	-	-/-	-	-/-	-	-/-	-	-/-	e	-	e	-	e	-	-	-/-

Ileal Peyer's patches	-	-/-	-	-/-	-	-/-	e	-/-	-	-/-	-	-/-	e	-	-	-	e*	-	e	-/-

	***Mycobacterium avium *subsp. *avium***																			

**Time after inoculation**	**6 weeks**										**12 weeks**									

**Animal no**	**#11**		**#12**		**#13**		**#14**		**#15**		**#16**		**#17**		**#18**		**#19**		**#20**	

	**gl**	**n/c**	**gl**	**n/c**	**gl**	**n/c**	**gl**	**n/c**	**gl**	**n/c**	**gl**	**n/c**	**gl**	**n/c**	**gl**	**n/c**	**gl**	**n/c**	**gl**	**n/c**

*Lnn. mandibulares*	e*	-/-	e	-/-	-	-/-	-	-/-	-	-/-	-	-/-	-	-/-	e	-/-	e*	-/-	e*	-/-

Tonsils	e	-/-	-	-/-	-	-/-	-	-/-	e	-/-	-	-/-	-	-/-	-	-/-	e*	-/-	e*	-/-

*Lnn. bifurcationis sinn*.	-	-/-	-	-/-	-	-/-	-	-/-	-	-/-	-	-/-	-	-/-	e	-/-	e	-/-	-	-/-

Lung	-	-/-	-	-/-	-	-/-	e	-/-	-	-/-	-	-/-	-	-/-	-	-/-	-	-/-	e	-/-

Spleen	e	-/-	-	-/-	-	-/-	-	-/-	-	-/-	-	-/-	-	-/-	-	-/-	-	-/-	-	-/-

Liver	-	-/-	-	-/-	-	-/-	-	-/-	-	-/-	-	-/-	-	-/-	-	-/-	e	-/-	-	-/-

*Lnn. jejunales*	-	-/-	-	-/-	-	-/-	-	-/-	-	-/-	-	-/-	-	-/-	e*	+/+	m*	+/+	e	-/-

*Lnn. ileocolici*	e	-/-	-	-/-	-	-/-	-	-/-	-	-/-	-	-/-	-	-/-	e	-	m	+/-	-	-/-

*Lnn. colici*	-	-/-	-	-/-	e	-/-	-	-/-	-	-/-	-	-/-	-	-/-	-	-	-	-	-	-/-

Jejunal Peyer's patches	-	-/-	-	-/-	e	-/-	-	-/-	-	-/-	-	-/-	-	-/-	e	-	e'	-	-	-/-

Ileal Peyer's patches	-	-/-	e*	-/-	-	-/-	-	-/-	-	-/-	e	-/-	-	-/-	e	-	e'	-	e*	-/-

*hominissuis *or *Mycobacterium avium *subsp. *avium*

gl = granulomatous lesion type

n = necrosis, c = calcification

e = epitheloid lesion

m = mixed lesion

* = acid-fast bacilli are present

' = ulcerative changes are present

Histopathological lesions were seen in every type of organ examined, but most frequently in gastrointestinal lymph nodes, *Lnn. mandibulares*, ileal Peyer's patches, tonsils, and jejunal Peyer's patches (Table 2). Lesions were found in 43% of the gastrointestinal lymph node samples in the *Mah *group and 23% in the *Maa *group. Calcification and necrosis were seen in gastrointestinal lymph nodes 12 weeks after infection with both subspecies, seven in animals infected with *Mah *and three in the *Maa *group. With regards to *Lnn. mandibulares *and tonsils, the number of samples with histopathological lesions, differed marginally between the two groups. In total, nine samples of *Lnn. mandibulares *and seven tonsils had histopathological lesions. Necrosis was observed in one tonsil and two *Lnn. mandibulares *from the *Mah *group, of which one was calcified.

On three occasions multinucleated giant cells were observed in samples from un-inoculated animals, indicating early granuloma formation. However, acid-fast bacilli and PCV2 virus were not detected, and infection is not strictly required for these changes to arise [38]. There might be unspecific reasons for the presence of multinucleated giant cells.

### Bacteriological examination

After 6 weeks of infection, *Mah *was detected by culture from faecal samples from four out of five animals, while *Maa *was not detected in faecal samples from any animal (Tables 1 and 3). This difference between the groups proved to be significant (*P *≤ 0.05). *Mah *was also cultured from faeces originating from animal #6, which was sacrificed at week eight. Twelve weeks after infection, none of the faecal samples were positive on culture.

**Table 3:** Mycobacterial growth in tissues and faeces from *Mycobacterium avium *subsp. *hominissuis *or *Mycobacterium avium *subsp. *avium *inoculated pigs

*Mycobacterium avium *subsp. *hominissuis*										
**Time after inoculation**	**6 weeks**					**8 weeks**	**12 weeks**			

**Animal no**	**#1**	**#2**	**#3**	**#4**	**#5**	**#6**	**#7**	**#8**	**#9**	**#10**

*Lnn. mandibulares*	4	1	4	3	4	3	1	3	4	4

Tonsils	4	1	4	4	4	4	1	C	4	4

*Lnn. bifurcationis sinn*.	1	-	-	-	-	-	-	-	2	-

Lung	-	-	-	-	-	-	-	-	-	-

Spleen	-	-	-	-	-	-	-	-	-	-

Liver	-	-	-	-	-	-	-	-	-	-

*Lnn. jejunales*	4	4	4	4	-	2	4	4	4	4

*Lnn. ileocolici*	3	3	-	2	-	1	2	4	4	-

*Lnn. colici*	4	4	-	-	-	4	-	4	4	-

Jejunal Peyer's patches	1	-	-	4	2	-	-	-	-	-

Ileal Peyer's patches	4	1	2	2	-	3	-	-	-	-

Faeces	4	1	3	2	-	2	-	-	-	-

***Mycobacterium avium *subsp. *avium***										

**Time after inoculation**	**6 weeks**					**12 weeks**				

**Animal no**	**#11**	**#12**	**#13**	**#14**	**#15**	**#16**	**#17**	**#18**	**#19**	**#20**

*Lnn. mandibulares*	3	2	1	-	1	1	1	4	4	4

Tonsils	-	-	-	-	-	-	-	-	1	1

*Lnn. bifurcationis sinn*.	-	-	-	-	-	-	-	-	-	-

Lung	-	-	-	-	-	-	-	-	-	-

Spleen	-	-	-	-	-	-	-	-	-	-

Liver	-	-	-	-	-	-	-	-	-	-

*Lnn. jejunales*	4	2	1	2	1	-	1	4	4	2

*Lnn. ileocolici*	1	1	-	2	1	-	-	3	4	4

*Lnn. colici*	-	1	-	-	1	1	2	-	-	2

Jejunal Peyer's patches	4	2	1	-	3	-	-	-	-	-

Ileal Peyer's patches	3	2	3	-	3	1	-	1	-	1

Faeces	-	-	-	-	-	-	-	-	-	-

1 = 1-9CFU, 2 = 10 - 49CFU, 3 = 50 - 100CFU, 4 = > 100CFU

C = contaminated

Mycobacteria were retrieved by culture from each of the infected animals, showing different tissues involved (Tables 1 and 3). In total, 50 organ samples from pigs infected with *Mah *and 43 samples from the *Maa *infected pigs contained live mycobacteria. IS*1245 *RFLP, IS*901 *PCR and IS*1245 *PCR confirmed that the isolated mycobacteria were of the same subspecies as the isolates used for inoculation, and that the RFLP profiles were unchanged. The distribution of positive samples was similar between the groups of subspecies and duration of infection. At 6 weeks of infection, the number of culture positive samples per animal of the *Mah *group ranged from three to eight out of 11, and in the *Maa *group from two to six. At 12 weeks, the range of positive samples per animal was three to six in both groups. Although not significantly different, there is a tendency of higher CFU counts in the samples from pigs inoculated with *Mah*, compared to *Maa*. More than 100 CFU were detected in 29 tissue samples from *Mah *infected pigs and in nine from the *Maa *group. Only eight samples from *Mah *infected pigs contained one to nine CFU, compared to 19 in the *Maa *group (Table 3).

All *Lnn. mandibulares*, except from one animal infected with *Maa*, were positive on culture. All tonsils from the animals in the *Mah *group were positive, except from one sample that was unreadable due to contamination by other bacteria. Only two tonsil samples were positive in the *Maa *group, both collected at 12 weeks (Tables 1 and 3). In total, the difference between bacterial growth from tonsil samples was significant between the two subspecies.

Live mycobacteria were detected in the gastrointestinal lymph nodes in all but one animal, which belonged to the *Mah *group sacrificed at 6 weeks. Bacteria were most frequently found in the jejunal lymph nodes. In addition, seven out of ten samples from jejunal or ileal Peyer's patches were positive on culture 6 weeks after infection in the *Mah *group. However, in the *Mah *group sacrificed at 12 weeks, all samples from the Peyer's patches were negative. In the *Maa *group sacrificed at 6 weeks, eight out of ten samples from Peyer's patches were positive, dropping to three out of ten after 12 weeks.

Mycobacteria were not found by culture from lungs, liver and spleen. However, live mycobacteria were detected in *Lnn. bifurcationis sinn*. in two animals of the *Mah *group, one sacrificed at 6 weeks and the other at 12 weeks.

DNA containing IS*1245 *was detected in tissue samples from all inoculated individuals by real time PCR (Table 1).

## Discussion

Infection with the *M. avium *subspecies was established in all inoculated animals, as shown by positive results in several of the analyses performed. This indicated that both *Mah *and *Maa *had the potential to establish infection in pigs, and once infected with either subspecies, the course of infection seemed to be quite similar. There was a significant difference between the animals infected with *Maa *and those infected with *Mah *with regards to faecal shedding. Whereas live *Mah *was profoundly excreted in faeces 6 weeks after inoculation, *Maa *was not detected in faecal samples. Although earlier events of shedding in the *Maa *infected pigs cannot be excluded, this observation points at a greater ability of *Mah *to cause an increased load of mycobacteria in the environment and an intensified infection pressure in the pig herd. Faecal shedding of *M. avium *in pigs has been described in the older literature by various authors [23-25]. Jørgensen et al. [23] demonstrated this event in the *Maa *strain ATCC 25291, with subsequent infection of contact animals. However, the following lack of excretion of mycobacteria from the contact animals led the authors to generally reject a faecal-oral route of transmission for *M. avium*. It is important to consider the fact that Jørgensen et al. used a laboratory strain whose properties might have been modified by numerous passages, and that the study involved *Maa *only. In the present study, the animals were inoculated with clinical isolates of *Maa *and *Mah *at comparable doses and kept under the same conditions, enabling the comparison between the subspecies.

In 1964, Kauker and Zettl [26] performed a comparative study with experimental infection in pigs using one mycobacterial isolate from a porcine lymph node and another from a bird, presumably *Mah *and *Maa*, respectively. Infection of contact animals was only seen in the group infected with the pig isolate, something that supports the hypothesis of differences between *Mah *and *Maa *with regards to transmission through faecal shedding. However, more recent papers postulate that *Mah *is not transmitted between pigs, due to the diversity of IS*1245 *RFLP profiles of isolates originating from the same farm [10,17]. Isolates of identical RFLP profiles have indeed been detected in pigs bred at the same facility [14,15,20]. The current findings propose that the route of faecal-oral infection in pigs is more pronounced in *Mah *than in *Maa*, which might be one reason for the higher incidence of the prior subspecies in the porcine population. One might hypothesize that *Maa *infection in pigs mainly occurs as individual events through ingestion of material containing mycobacteria, such as bird shedding or peat [7], whereas *Mah *additionally has a greater ability to keep circulating in the pig herd once introduced. Additionally, *Mah *is proposed to be the only true environmental subspecies of *M. avium *[2], suggesting a better ability of *Mah *than *Maa *to replicate in the environment. There is no doubt that *Mah *can be introduced to pig herds by contaminated peat and sawdust, still transmission from pig to pig also seems to occur.

The most obvious finding of the present study with regards to differences in the count of live bacteria in organs was done in tonsils, where *Mah *proved to be present in considerable amounts, while *Maa *was only sporadically detected. The tonsils are a proposed reservoir for the faecal-oral route of transmission [25] and strengthens the hypothesis of *Mah *being more likely than *Maa *to spread by pig-to-pig contact.

The amount of mycobacteria and the numbers and severity of tuberculous lesions detected in the present study was somewhat greater in pigs inoculated with *Mah *than with *Maa*, although not significantly. This seems to be in contrast to the study of Kauker and Zettl [26], who found the bird isolate to be hypervirulent, causing systemic disease and death, while the pig isolate only gave rise to lymph node lesions. One should bear in mind that Kauker and Zettl had only two infected animals and one control- and contact animal per group, which makes it difficult to draw any conclusions.

The extent of intracellular replication in cell cultures is widely used as a measure for mycobacterial virulence [39-41]. Results from the present infection assays in porcine macrophage cultures suggested that *Maa *replicated intracellularly to a greater extent than *Mah*, thus defining it as more virulent by the prevailing standards. The results from the cell assay support the assumption that the low prevalence of mycobacteriosis caused by *Maa *in the pig population is not due to lack of virulence or failure to establish infection, which was confirmed by the experimental infection in pigs. However, the contradictory results of the present in vitro and in vivo studies, suggests that assaying virulence of facultative pathogen mycobacteria based only on in vitro studies should be done with some caution.

In the present study, mycobacteria were mainly detected in lymph nodes, tonsils and intestinal segments, which have previously been reported as typical locations [4]. In concordance with other studies [10,18], massive occurrence of live mycobacteria and exudative histopathological lesions not accompanied by gross lesions were findings in a majority of samples. In *Lnn. mandibulares*, macroscopic lesions characteristic of mycobacterial infection were only detected in two of the pigs, even though mycobacteria could be detected by culture from various organs in all pigs. This should be of interest with regards to food safety, as the gross examination of *Lnn. mandibulares *plays an important role in the detection of mycobacterial infection in slaughtered pigs.

The present study indicated that *Maa *has an infection potential in pigs on almost the same level as *Mah*, leaving differences in exposure or sensitivity of detection at slaughter as probable reasons for the fact that *Maa *has not been reported in the Norwegian swine population. In some countries, *Maa *has been detected in pigs [7,8], something that might be explained by differences in housing. Pig housing that allows birds to enter, or the keeping of free range pigs, presumably enables an increased infection pressure of *Maa*. In Norway, pig farms are mainly closed in-door facilities, and *Maa *infections in the Norwegian bird population are rarely diagnosed (B. Djønne, NVI, personal communication). *Maa *has also sporadically been detected in peat [18], which leaves regional differences in peat processing as an additional explanation for the fact that some countries are more troubled with *Maa *infection in pigs than others [7].

## Conclusions

The present study re-introduces a hypothesis of a faecal-oral route of infection being a more prominent feature in *Mah *than in *Maa*. This might contribute to the fact that *Mah *is the more commonly detected subspecies in pigs. Additionally, low exposure to infected birds might explain the absence of *Maa *infection in pigs in many countries. The observations of the present study also propose that the higher reported incidence of infection with *Mah *than *Maa *in the pig population might be due to the formation of more obvious lesions in the prior. Nevertheless, future assays of experimental infection with contact animals of young age and other clinical isolates of *Maa *and *Mah *are needed to mimic the situation in swine farms and to conclude on behalf of the subspecies with regards to different strategies of transmission and virulence.

## Competing interests

The authors state that there are no competing interests related to the present study.

## Authors' contributions

AA contributed to conception and design of the experiment, sampling and bacteriological analysis, data analysis and drafting of the manuscript. TBJ contributed to conception and design of the experiment, sampling and bacteriological analysis and drafting of the manuscript. ØK contributed to necropsy and histopathological examination and writing of the manuscript. AJ contributed with advice on pig husbandry and to collection of blood samples and writing of the manuscript. BD contributed to conception and design, bacteriological analysis and drafting of the manuscript. IO contributed to conception and design of the experiment, sampling, immunological analysis and drafting of the manuscript. All authors read and approved the final manuscript.
